# Ovarian tissue cryopreservation and transplantation: 20 years experience in Bologna University

**DOI:** 10.3389/fendo.2022.1035109

**Published:** 2022-10-12

**Authors:** Raffaella Fabbri, Rossella Vicenti, Valentina Magnani, Roberto Paradisi, Mario Lima, Lucia De Meis, Stefania Rossi, Diego Raimondo, Paolo Casadio, Stefano Venturoli, Michela Maffi, Renato Seracchioli

**Affiliations:** ^1^ Department of Medical and Surgical Sciences, University of Bologna, Bologna, Italy; ^2^ Division of Gynaecology and Human Reproduction Physiopathology, IRCCS Azienda Ospedaliero-Universitaria di Bologna, Bologna, Italy; ^3^ Pediatric Surgery Department, IRCCS Azienda Ospedaliero-Universitaria di Bologna, Bologna, Italy

**Keywords:** fertility preservation, ovarian tissue cryopreservation, ovarian tissue transplantation, cancer, laparoscopy

## Abstract

**Objective:**

To report the 20-year experience in ovarian tissue cryopreservation (OTC) and ovarian tissue transplantation (OTT) of the Bologna clinical center (Bologna, Italy).

**Design:**

Retrospective cohort study.

**Patients:**

1026 pediatrics and women aged between 2 and 38 years who underwent OTC and OTT between January 2002 to January 2022.

**Results:**

Of the 1026 patients, 238 (22.8%) were pediatrics (≤ 17 years, Group 1) and 788 (77.2%) were adult women (range 18-38 years, Group 2). In Group 1, 184 (77.3%) patients had malignant diseases and 54 (22.7%) had non-malignant diseases. In Group 2, 746 (94.7%) patients had malignant diseases and 42 (5.3%) had non-malignant diseases. No real complications were observed during surgery. In all the samples analyzed most of the follicles were in the resting stage, while only a few follicles were growing. In both fresh and thawed samples, follicular density was higher in Group 1 than in Group 2 (p < 0.01). Regardless of age, good preservation of follicles and stroma was observed in fresh and thawed ovarian tissue by histological and immunohistochemical analyses (estrogen and progesterone receptors; Ki67 and Bcl2 markers; TUNEL). To date, out of 1026 total women, 812 (79.1%) had their tissue stored. Sixty-eight (6.6%) patients died from their primary disease. Twenty-four (2.3%) women performed 33 OTTs between December 2011 and January 2022. Restoration of menstruation was observed in 15 out of 17 menopausal women. Six pregnancies were achieved, two hesitated in abortion and four in the birth of healthy babies.

**Conclusion:**

OTC is the only fertility preservation technique applicable in pre-pubertal/pediatrics and in adult patients when stimulation for oocytes/embryos cryopreservation is not possible. The reported data can help future patients and physicians in their discussions and decisions about the need and possibilities of preserving ovarian function.

## Introduction

Ovarian tissue cryopreservation (OTC) and transplantation (OTT) with > 200 children born worldwide is a valid strategy to preserve endocrine and reproductive function in pediatric and adult women at high risk of premature ovarian insufficiency (POI). OTC benefits not only women with oncological diseases, but also those with autoimmune diseases requiring bone marrow transplantation or with systemic (B-thalassemia) or local (endometriosis) benign diseases, or with genetic diseases (1). After remission, cryopreserved ovarian tissue can be transplant in women, allowing the recovery of ovarian function and spontaneous pregnancy. Transplant efficiency was established as ovarian function recovery (95% of cases), number of live births (37% live births/transplantation number) (2–9) and induction of puberty (10–12). Unfortunately, the lack of official international registries and the lack of reported results in many centers lead to poor knowledge of the outcomes after OTC and OTT (1).

The aim of the study is to describe the casuistry of 20 years of experience in pediatric and women undergoing OTC to preserve ovarian function and fertility at the Bologna clinical center (Bologna, Italy).

## Material and methods

### Subjects and preoperative evaluation

This is a retrospective study including pediatrics and women aged between 2 and 38 years who underwent OTC between January 2002 to January 2022 at our center, to preserve ovarian function due to an oncological, hematological, or other pathology (genetic or autoimmune diseases). Within a period of 24-36 hours before OTC, all patients, on a random day of their menstrual cycle, underwent hormonal assay for follicle stimulating hormone (FSH), luteinizing hormone (LH), prolactin (PRL), estrone (E_1_), Estradiol (E_2_), 17-hydroxyprogesterone, inhibin B (In-B), anti antimüllerian hormone (AMH) and ultrasound investigation for some ovarian markers, i.e. mean ovarian volume, antral follicle count (AFC), presence of corpus luteum or dominant follicle. Inclusion criteria for enrollment were: no previous pelvic surgery, no perimenopausal conditions identified by FSH and LH levels, age-matched normal ultrasonographic markers, no abnormalities in ovarian morphology, no evidence of endocrine/metabolic or systemic diseases (13). To ensure greater safety of the entire course of OTC and OTT procedures the patients were evaluated by the specialist team of our center, consisting of gynecologists, endocrinologists, biologists, pediatric surgeons, anesthesiologists, nurses, and psychologists. The OTC, OTT and patients’ data collection were approved by our local Ethics Committee (N. 74/2001/O). An informed written consent was signed by all patients, and, in the case of minors, by their parents.

### Ovarian tissue cryopreservation

A large ovarian biopsy, approximately 30-40% of the ovary from one or both ovaries, was obtained by laparoscopy as a daytime surgical procedure under general anesthesia, taking care to avoid damage to the ovarian tissue (with the use of Endobag), according to the technique of Paradisi et al. (14). To ensure safe surgery for pediatric patients the surgery was performed according to the procedure of Lima et al. (15). The harvested tissue was then immediately transferred to the laboratory in fresh phosphate-buffered saline medium (PBS) supplemented with 10% human serum (HS), provided by our Transfusion Centre, on ice. The cortical tissue was dissected into strips (± 10 × 2 × 2mm) and frozen slowly according to the protocol described by Fabbri et al. (16). Briefly, the ovarian cortical slices were placed in precooled plastic cryovials (Intermed Nunc Cryotubes) containing 1.8 mL of the freezing solution, and were transferred to a rolling system for 1 hour at 4°C. The cryovials were cooled in a programmable freezer (Planer Kryo 10/1.7 Series III; Sapio Life) and then transferred into liquid nitrogen until ready to be thawed.

A cortical piece of ovarian tissue (± 2 × 2 × 2 mm) for each patient was thawed according to Fabbri et al. (17) about a month after OTC to perform a qualitative control analysis of the freezing procedure. To thaw, the vials were air warmed for 30 seconds then immersed in a 37°C water bath for 2 minutes. The cryoprotectants were removed at 4°C by their stepwise dilution in the thawing solution. Finally, the tissue pieces were incubated for 20 minutes in a solution of DPBS supplemented with 30% HS at room temperature.

### Qualitative and functional analysis of fresh and thawed ovarian tissue

Fresh and frozen/thawed tissue samples were fixed in a freshly prepared solution of 2.5% glutaraldehyde in 0.1 mol/L sodium cacodylate buffer and processed as already described (18). For each sample, 0.5 μm thick section out of 50 μm of fixed tissue was collected and stained with toluidine blue for light microscopy (LM) examination (LeitzDiaplan light microscope equipped with a CCD JVC camera). Double blind semithin sections were examined by two trained pathologists, to (i) identify and count the follicles, (ii) determine the percentage of damaged follicles, and (iii) assess follicular density, expressed as the number of follicles per square millimeter of the overall section area (n/mm^2^) (18). The follicles were counted and classified according to Gougeon classification (19). The quality of follicles was determined. Follicles containing oocytes with empty appearance, large cytoplasmic vacuoles, dark and granular cytoplasm, hyperchromatic nuclear staining, and detachment of the oocyte from granulosa cells were considered degenerated.

Fresh and frozen/thawed tissue samples were analyzed according to Fabbri et al. (17). Briefly, the strips were embedded in paraffin blocks and nine serial sections 4 μm thickness per block were obtained: the first, third and seventh were stained with hematoxylin and eosin (Merck, Darmstadt, Germany), to assess the morphological characteristics of follicles and stromal cells. The second, fourth and sixth sections were used for immunohistochemical analysis: anti-estrogen receptor clone 1D5 (1:20, Bio Genex, San Ramon, CA), anti-progesterone receptor clone 1A6 (1:20, Bio Genex, San Ramon, CA), anti-proliferating antigen Ki67 (1:80, Bio Genex, San Ramon, CA), anti-protein Bcl2 (1:80, Dako, Carpinteria, CA). The fifth, eighth and ninth sections were collected (Superfrost-plus slides Menzel-Gläser, Braunschweig, Germany) for the analysis of the terminal deoxynucleotidyl transferase dUTP nick end labeling (TUNEL) using the cell death detection kit combined with horseradish peroxidase (Roche, Mannheim, Germany), according to the manufacturer’s instructions.

### Ovarian tissue transplantation

The transplants were carried out after the cancer treatment was completed and after a remission period of at least 2 years. The tissue was transplanted into women who had POI and also those women without POI who did not get pregnant spontaneously and required transplantation to increase the chances of pregnancy.

Before the transplant, the treating oncologist/hematologist was contacted for approval and a small fragment of ovarian tissue was analyzed to detect the presence of micrometastases using different histological, immunohistochemical and molecular methods depending on the patient’s primary pathology. In addition, the couple underwent infertility screening to rule out or correct other potential causes of infertility, in particular a check of the patency of the Fallopian tubes by sonosalpingography and an evaluation of the uterine cavity by hysteroscopy. An analysis of the partner’s semen was also performed before the transplant.

The orthotopic and heterotopic transplantation procedures were already described in our previous study (20). For heterotopic transplantation the cortical strips were gently placed in subcutaneous pockets created above the fascia in the suprapubic area and closed with a 4-0 Vicryl suture. Orthotopic transplant was performed using four-port laparoscopy. A longitudinal incision of approximately 1 cm was made on the surface of the residual ovaries and a pocket was developed in the ovarian parenchyma by blunt dissection. Cortical strips were sutured into the ovarian pocket and the same sutures were used to close the ovary, taking care not to cause tissue ischemia. Some strips were also inserted into a peritoneal pocket close to the ovary and then closed with a 4-0 Vicryl suture.

### Statistical analysis

Statistical analyses were performed by using the Statistical Program for Social Sciences (IBM SPSS, version 21.0, IBM Co., Armonk, New York, United States). Absence of normality distribution of quantitative variables was assessed using Kolmogorov-Smirnov test. Nonparametric statistics for quantitative variables were then applied and the Mann-Whitney U test was used to determine differences between the groups. Two-tailed P values <.05 were considered statistically significant.

## Results

### Age and diagnoses at OTC time

**Figure 1:**
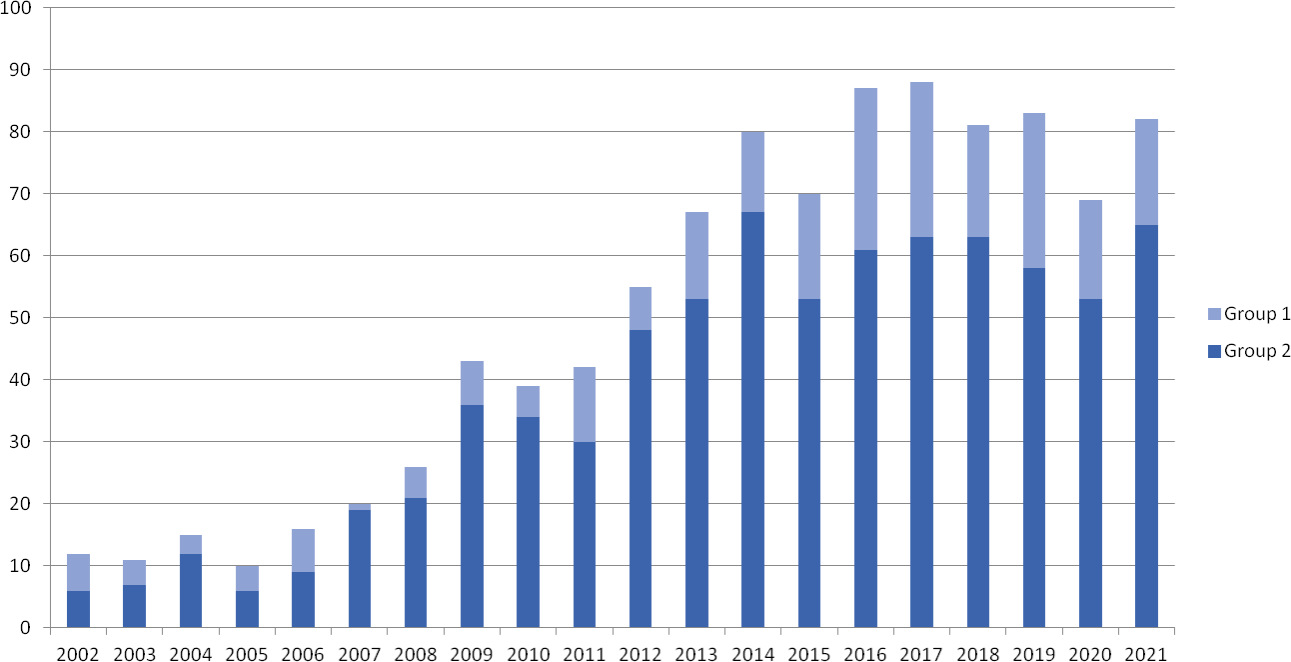
Ovarian tissue cryopreservation per year. Representative histogram of cryopreservations performed in Group 1 (pediatric girls, ≤ 17 years) and Group 2 (adult women, range 18-38 years) per year from 2002 to 2021.

Between January 2002 and January 2022, 1026 female patients underwent OTC for fertility preservation at our center. The number of OTCs executed per years has steadily increased until 2014, after which it stabilized at approximately 80 cases per year (**Figure 1**). Of the 1026 patients, 238 (23.2%) were pediatrics (≤ 17 years, Group 1) and 788 (76.8%) were adult women (range 18-38 years, Group 2). The mean ± SD age was 12.9 ± 4.14 years in Group 1 and 28.0 ± 5.7 years in Group 2.

In **Table 1** the types, numbers and percentages of diagnoses were reported for the overall patients analyzed in the study and dividing the patients in Group 1 and Group 2. Overall, the malignant diseases have a higher incidence than non-malignant diseases. In the malignant diseases’ subgroup, the lymphomas being the most frequent finding in both Group 1 and 2, followed by sarcomas in Group 1and breast cancer in Group 2. While in the non-malignant diseases’ subgroup genetic and autoimmune diseases are equally distributed between Group 1 and Group 2 (**Table 1**).

**Table 1:** Type of diagnosis in Group 1 and 2.

		Diseases	Overall (Group 1+Group 2)	Group 1	Group 2
Malignant Diseases		lymphomas	419	82	337
		leukemias	30	10	20
		myelodisplasias	13	0	13
		breast cancers	260	0	260
		sarcomas	101	49	52
		neurological malignancies	39	13	26
		gastrointestinal malignancies	26	15	11
		gynecologic malignancies	22	0	22
		Wilms tumors	9	9	0
		Others	11	6	5
		**TOTAL (Malignant Diseases)**	**930**	**184**	**746**
Non-malignant diseases		genetic diseases	52	38	14
		autoimmune diseases	17	7	10
		others	27	9	18
		**TOTAL (Non-Malignant Diseases)**	**96**	**54**	**42**
	**TOTAL (Malignant and Non-Malignant Diseases)**		**1026**	**238**	**788**

Types, numbers and percentages of the different pathologies of the 1026 patients divided into Group 1 (pediatric girls, ≤ 17 years) and Group 2 (adult women, range 18-38 years).

Regarding the distribution of pathologies in relation to age represented by the Group 1 and 2, more than 87% of patients with breast cancers, hematological diseases, gastrointestinal diseases, gynecological diseases were >17 years of age at OTC, whereas the most patients (77%) with genetic diseases and Wilms tumors were <17 years of age. Pathologies such as sarcomas, autoimmune diseases, gastrointestinal diseases were equally distributed in the two groups analyzed.

Two hundred twenty-six (22%) patients (n=70 Group 1; n=156 Group 2) underwent OTC after having already started chemotherapy (one or two cycles). All patients still had signs of ovarian activity (AMH > 1,5 ng/ml, FSH < 15 mU/ml) and presence of antral follicles. Pre-OTC chemotherapy treatments were found prevalent for systemic tumors in lymphomas (29 in Group 1 and 102 in Group 2) and leukemia malignancies (9 in Group 1 and 20 in Group 2); while for solid tumors in sarcomas (23 in Group 1 and 13 in Group 2) and breast cancers (9 cancers in Group 1).

### Surgical outcomes and complication of laparoscopy

No patients received blood transfusion during or after surgery and no real complications were observed. All Group 1 patients were discharged 2 days after surgery for added safety; while all Group 2 patients were discharged the day after surgery. Most patients started anti-cancer treatment within 7 days after OTC, except for patient with thalassemia major who were not emergency cases.

### Qualitative and functional analysis of fresh and thawed ovarian tissue

#### Histological Analysis

**Figure 2:**
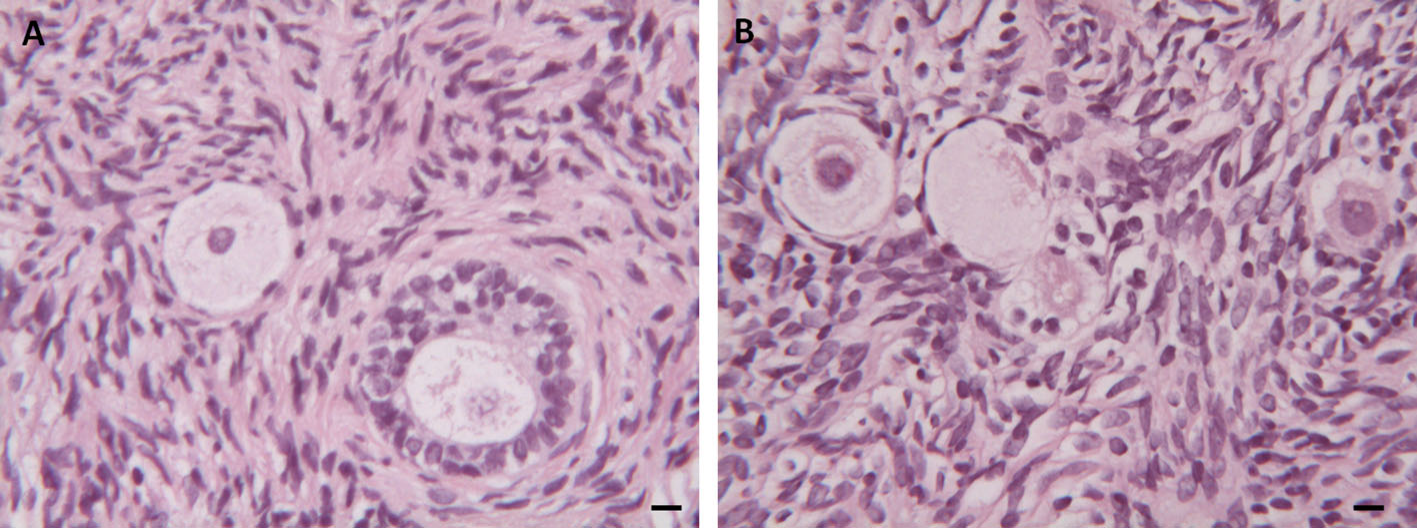
Histological Analysis. **(A)** Morphological appearance of follicles and stroma in fresh ovarian tissue. **(B)** Mild interstitial edema, widespread vacuolization and chromatin in thawed ovarian samples. Scale Bar 25 µm.

The effects of the cryopreservation procedure on stromal structure and follicular preservation and density were evaluated by light microscopy (LM) in 270 randomly selected patients. Homogeneous distribution of stromal cells with regular nuclei in fresh and thawed samples was observed in both groups (**Figure 2A**). Mild interstitial edema, widespread vacuolization and marked chromatin clumping were found in the thawed samples of both Groups (**Figure 2B**). In all the samples analyzed most of the follicles were in the resting stage, while only a few follicles were growing (3% in Group 1 and 4% in Group 2). Follicular densities were higher in Group 1 than in Group 2 (127.2 vs 19.1 n/mm^2^; p < 0.01). In addition, the same outcome was observed in both groups regardless of the number of total follicles in mm^2^ when comparing fresh and thawed samples (p < 0.01). No differences were found when analyzing the percentage of damaged follicles in both fresh and thawed tissues in the two groups (p = 0.12, NS).

#### Immunohistochemical analysis

**Figure 3:**
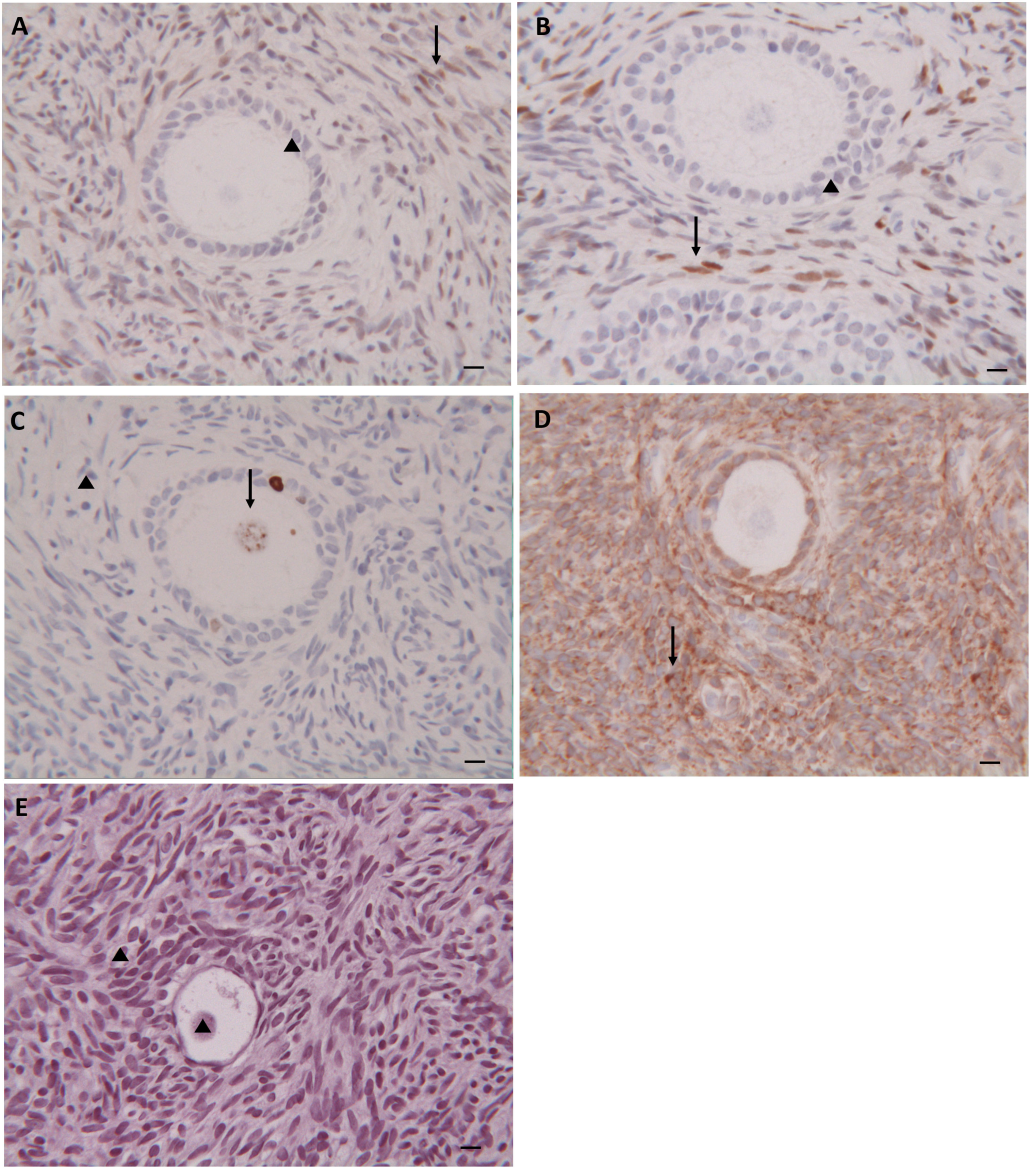
Immunohistochemical Analysis. Immunohistochemical staining for estogen **(A)**, progesterone **(B)**, ki-67 **(C)**, bcl2 **(D)** and TUNEL **(E)** in follicles and stroma of thawed ovarian tissue. (▲) Negative staining; (↑) Positive staining. Scale Bar 25 µm.

Immunohistochemical analysis, performed in the same 270 randomly selected patients, showed similar staining pattern distribution in both groups and both before and after cryopreservation. In particular, estrogen receptors were negative in all follicles examined and showed patch/focal expression in stromal cells (**Figure 3A**). Progesterone receptors were negative in the follicles but diffusely positive in the stromal cells (**Figure 3B**). Positive nuclear staining for the Ki67 protein was found in both granulosa cells and/or oocytes, while no positive staining was observed in stromal tissue (**Figure 3C**). Positive staining for the bcl2 protein was found in granulosa cells of secondary/preantral follicles, but not in oocytes. Stromal tissue was diffusely stained with bcl2 (**Figure 3D**). No TUNEL-positive cells were found in the primordial follicles and in those at more advanced stage of development, as well as in the stroma (**Figure 3E**).

### State of stored ovarian tissue

Every year women and parents, in the case of minors, renew the desire to keep their ovarian tissues cryopreserved. To date, out a total of 1026 women, 812 (79.1%) had their tissue stored: 280 of them (34.5%) were aged <30 years (22 ± 5.80, mean ± SD age), 427 (52.6%) were aged between 30 and 40 years (35 ± 3.14, mean ± SD age) and 105 (12.9%) were aged >40 years (45 ± 4.34, mean ± SD age). One hundred and eighty (17.6%) patients donated their tissue for research, 10 (1%) decided to destroy their own tissue and 24 (2.3%) reimplanted all their ovarian tissue. Out of 180 tissues donated, 68 women died of primary disease (breast cancer, sarcomas, hematological diseases – 94%) during the follow-up. The remaining 112 women who donated ovarian tissue had a prevalence of systemic diseases (leukemia and lymphoma – 17%).

Twenty-four women performed 33 OTTs between December 2011 and January 2022. The types of diagnosis were distributed as follows: 9 patients had breast cancers, 8 hematological diseases, 2 sarcomas, 1 medulloblastoma, 1 struma ovari, 2 colon cancers and 1 cervical cancer. At the time of OTT, 17 women were in POI, and the remaining 7 women were still having irregular menses and difficult to become pregnant. Orthotopic transplantations were performed in 20 women (28OTTs) to restore and/or improve ovarian function and seek pregnancy; while heterotopic transplantations were performed in 4 women (5 OTTs) to restore steroidogenesis. The mean ± SD age of the transplanted women was 29.6 ± 7.3 at the time of OTC, 36.24 ± 5.9 at the time of first orthotopic transplantation and 33.75 ± 6.2 at the time of first heterotopic transplantation. The mean storage time was 7.84 ± 3.5 years (range 2-17 years).

In the group of POI women (17 women), restoration of menstruation was achieved in 11 (85%) of the 13 women who performed orthotopic transplantation and in all 4 (100%) women who performed heterotopic transplantation. Six pregnancies were achieved, two hesitated in abortion and four in the birth of healthy babies (3 spontaneous and 1 IVF-inducted pregnancy).

Regarding relapses, a woman healing from Ewing sarcoma had a recurrence of the disease five months after OTT and she performed additional chemotherapy. At the time of analysis all transplanted women are in good health.

### Mortality

Sixtyeight (6.6%) patients died from their primary disease (Group 1 = 27; Group 2 = 41). According to the diagnosis, in Group 1 the highest relative mortality was sarcomas (38%), hematological diseases (30%) and genetic diseases (15%); in Group 2 the highest relative mortality was breast cancer (59%), hematological diseases (48%) and sarcomas (13%). While the interval between OTC and death in Group 2 was 5 ± 3 years for patient with breast cancers and 1 ± 1 year (mean ± SD) for patients with hematological diseases. The interval between OTC and death was very short in Group 1, resulting in a maximum of 1 or 2 years.

## Discussion

These data place our center as the Italian reference center by numbers of patients for the cryopreservation and transplantation of ovarian tissue. Many European hospitals and fertility centers have included OTC in their fertility preservation programs alongside commonly used methods, with about 23 ovarian tissue banks distributed in these countries (21).

Methods for female fertility preservation have been developed in recent decades and are currently represented by the cryopreservation of embryos and oocytes. The OTC is still considered an experimental procedure internationally. However, in many countries (USA, Israel, Denmark, Norway, and France) OTC is now regarded as a consolidated and standardized technique for fertility preservation (22).

Furthermore, to date OTC and OTT procedures are considered safe for both adults and children (23). It is estimated that the occurrence of severe complications is arising from surgery appear to be rare and, in any case, less than 1% (24, 25). According to the literature, no patients in our study experienced postoperative bleeding requiring a reoperation. Transplant efficiency was established as ovarian function recovery (95% of cases), number of live births (37% live births/transplantation number) (2–9) and induction of puberty (10–12). Our outcomes are in agreement with literature data. In this study we report 33 OTTs in 24 women, with 2% of transplantation rate. In the group of POI women, the restoration of ovarian function was 100% after heterotopic transplantation (4 patients) and 85% after orthotopic transplantation (11 women); the live births rate was 67% (4 live births and 2 miscarriage).

OTC is recommended for women in whom the preservation of ovarian function is directly or indirectly threatened due to an underlying oncological, hematological or other disease (26). This is especially true in the case of imminent gonadotoxic chemotherapy or radiotherapy in the lower pelvis. The OTC should be offered especially to all women at a high and intermediate risk of POI. However, even in women with low risk of POI, this option should be discussed with the patient. In fact, the risk of POI as a long-term complication associated with cancer treatments is more common than previously estimated and can occur even many years after treatments (24).

Moreover, OTC is the only option for pre-pubertal girls requiring gonadotoxic treatment, although access to this procedure in these patients is still limited. As reported by various authors (6, 10, 27), this is partly due to the fact that this procedure continues to be considered experimental, and therefore not assured. Usually, the ovarian tissue of these patients remains frozen for a long period of time. We recently demonstrated that ovarian tissue stored for 18 years maintained its morpho-functional integrity (17). In addition, we recently reported ovarian function recovery after heterotopic OTT stored for 11 and 15 years, providing strong evidence that time has no impact on tissue preservation and ovarian function recovery (28).

It is therefore generally recommended that ovarian tissue can be cryopreserved prior to initiation of chemotherapy (29). Nevertheless, some clinical conditions prove the exception to the rule, such as patients with acute leukemia in whom OTC cannot be performed before the start of chemotherapy. In these circumstances, the recommendations of the European Society of Human Reproduction and Embryology state that, “patients who have already received low gonadotoxic treatment or a previous course of chemotherapy can be offered OTC as a fertility preservation option” (30). This is in agreement with recent studies showing that exposure to chemotherapy before OTC does not alter OTT outcomes and live birth rates (3, 5, 30, 31). However, caution is advised when alkylating agents are administered prior to OTC as several studies demonstrated the harmful effects of these agents on reimplantation outcomes (29, 30, 32).

The field of fertility preservation is constantly evolving. There is an ethical obligation for physicians to provide information on the impact of cancer treatment on future fertility and to discuss fertility issues with patients in order to preserve the chances of future parenthood. Our data can help future patients and physicians in their discussions and decisions about the need and possibilities to preserve ovarian function. This will lead to an increase in the efficiency and applicability of care.

## Data availability statement

The raw data supporting the conclusions of this article will be made available by the authors, without undue reservation.

## Ethics statement

The studies involving human participants were reviewed and approved by N. 74/2001/O. Written informed consent to participate in this study was provided by the participants’ legal guardian/next of kin.

## Author contributions

RF, RS, and SV planned the study. RP, ML, DR, PC, SV, and MM performed ovarian tissue retrieved. RF, RV, and VM cryopreserved and thawed ovarian tissue. RS, RP, DR, PC, and SV performed ovarian tissue transplantation. RV, VM, RP, and RF analyzed, collected data, and drafted the paper. LM and SR performed gynecologic and obstetrics follow-up of the patients. All authors contributed to the article and approved the submitted version.

## Funding

The work reported in this publication was funded by the Italian Ministry of Health, RC-2022-2773473 project.

## Acknowledgments

The authors thank all personnel, including those engaged in the clinical and laboratory activities in our fertility preservation program.

## Conflict of interest

The authors declare that the research was conducted in the absence of any commercial or financial relationships that could be construed as a potential conflict of interest.

## Publisher’s note

All claims expressed in this article are solely those of the authors and do not necessarily represent those of their affiliated organizations, or those of the publisher, the editors and the reviewers. Any product that may be evaluated in this article, or claim that may be made by its manufacturer, is not guaranteed or endorsed by the publisher.
