# Corrigendum: Ovarian tissue cryopreservation and transplantation: 20 years experience in Bologna University

**DOI:** 10.3389/fendo.2022.1095627

**Published:** 2022-11-25

**Authors:** Raffaella Fabbri, Rossella Vicenti, Valentina Magnani, Roberto Paradisi, Mario Lima, Lucia De Meis, Stefania Rossi, Diego Raimondo, Paolo Casadio, Stefano Venturoli, Michela Maffi, Renato Seracchioli

**Affiliations:** ^1^ Department of Medical and Surgical Sciences, University of Bologna, Bologna, Italy; ^2^ Division of Gynaecology and Human Reproduction Physiopathology, IRCCS Azienda Ospedaliero-Universitaria di Bologna, Bologna, Italy; ^3^ Pediatric Surgery Department, IRCCS Azienda Ospedaliero-Universitaria di Bologna, Bologna, Italy

**Keywords:** fertility preservation, ovarian tissue cryopreservation, ovarian tissue transplantation, cancer, laparoscopy

In the published article, there was an error in affiliation number 2. Instead of “Department of Medical and Surgical Sciences, Istituti di Ricovero e Cura a Carattere Scientifico (IRCCS) Azienda Ospedaliero-Universitaria di Bologna, Bologna, Italy”, it should be removed.

In the published article, there was an error in affiliation number 3. Instead of “Pediatric Surgery Department, Istituti di Ricovero e Cura a Carattere Scientifico (IRCCS) Azienda Ospedaliero-Universitaria di Bologna, Istituti di Ricovero e Cura a Carattere Scientifico (IRCCS), Bologna, Italy”, it should be “Pediatric Surgery Department, IRCCS Azienda Ospedaliero-Universitaria di Bologna, Bologna, Italy”.

In the published article, there was an error in affiliation number 4. Instead of “Division of Gynaecology and Human Reproduction Physiopathology, Istituti di Ricovero e Cura a Carattere Scientifico (IRCCS) Azienda Ospedaliero-Universitaria di Bologna, Istituti di Ricovero e Cura a Carattere Scientifico (IRCCS), Bologna, Italy”, it should be affiliation number 2 and changed as following “Division of Gynaecology and Human Reproduction Physiopathology, IRCCS Azienda Ospedaliero-Universitaria di Bologna, Bologna, Italy”.

In the published article, there was an error in affiliation number 5. Instead of “Department of Medical and Surgical Sciences, Division of Gynaecology and Human Reproduction Physiopathology, Istituti di Ricovero e Cura a Carattere Scientifico (IRCCS) Azienda Ospedaliero-Universitaria di Bologna, University of Bologna, Bologna, Italy”, it should be removed.

The authors apologize for these error sand state that they do not change the scientific conclusions of the article in any way. The original article has been updated.

## Publisher’s note

All claims expressed in this article are solely those of the authors and do not necessarily represent those of their affiliated organizations, or those of the publisher, the editors and the reviewers. Any product that may be evaluated in this article, or claim that may be made by its manufacturer, is not guaranteed or endorsed by the publisher.

